# Efficacy of compressed sensing and deep learning reconstruction for adult female pelvic MRI at 1.5 T

**DOI:** 10.1186/s41747-024-00506-5

**Published:** 2024-09-10

**Authors:** Takahiro Ueda, Kaori Yamamoto, Natsuka Yazawa, Ikki Tozawa, Masato Ikedo, Masao Yui, Hiroyuki Nagata, Masahiko Nomura, Yoshiyuki Ozawa, Yoshiharu Ohno

**Affiliations:** 1 Department of Diagnostic Radiology, Fujita Health University School of Medicine, Toyoake, Japan; 2 Canon Medical Systems Corporation, Otawara, Japan; 3 Department of Radiology, Fujita Health University Bantane Hospital, Nagoya, Japan; 4 Joint Research Laboratory of Advanced Medical Imaging, Fujita Health University School of Medicine, Toyoake, Japan

**Keywords:** Deep learning, Diagnostic imaging, Female, Magnetic resonance imaging, Pelvis

## Abstract

**Background:**

We aimed to determine the capabilities of compressed sensing (CS) and deep learning reconstruction (DLR) with those of conventional parallel imaging (PI) for improving image quality while reducing examination time on female pelvic 1.5-T magnetic resonance imaging (MRI).

**Methods:**

Fifty-two consecutive female patients with various pelvic diseases underwent MRI with T1- and T2-weighted sequences using CS and PI. All CS data was reconstructed with and without DLR. Signal-to-noise ratio (SNR) of muscle and contrast-to-noise ratio (CNR) between fat tissue and iliac muscle on T1-weighted images (T1WI) and between myometrium and straight muscle on T2-weighted images (T2WI) were determined through region-of-interest measurements. Overall image quality (OIQ) and diagnostic confidence level (DCL) were evaluated on 5-point scales. SNRs and CNRs were compared using Tukey’s test, and qualitative indexes using the Wilcoxon signed-rank test.

**Results:**

SNRs of T1WI and T2WI obtained using CS with DLR were higher than those using CS without DLR or conventional PI (*p* < 0.010). CNRs of T1WI and T2WI obtained using CS with DLR were higher than those using CS without DLR or conventional PI (*p* < 0.003). OIQ of T1WI and T2WI obtained using CS with DLR were higher than that using CS without DLR or conventional PI (*p* < 0.001). DCL of T2WI obtained using CS with DLR was higher than that using conventional PI or CS without DLR (*p* < 0.001).

**Conclusion:**

CS with DLR provided better image quality and shorter examination time than those obtainable with PI for female pelvic 1.5-T MRI.

**Relevance statement:**

CS with DLR can be considered effective for attaining better image quality and shorter examination time for female pelvic MRI at 1.5 T compared with those obtainable with PI.

**Key Points:**

- Patients underwent MRI with T1- and T2-weighted sequences using CS and PI.
- All CS data was reconstructed with and without DLR.
- CS with DLR allowed for examination times significantly shorter than those of PI and provided significantly higher signal- and CNRs, as well as OIQ.

**Graphical Abstract:**

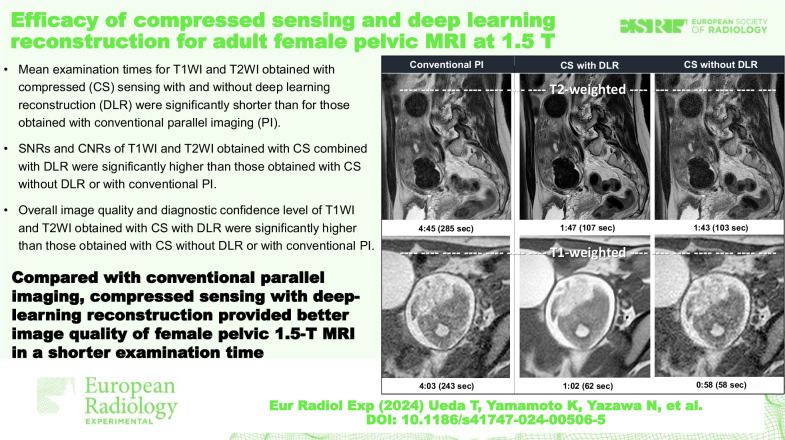

## Background

Magnetic resonance imaging (MRI) is widely accepted as an effective modality for evaluating female pelvic diseases, with T2-weighted sequences yielding excellent soft tissue contrast of the pelvic organ [1].

According to Organization for Economic Co-operation and Development (OECD) data, MRI examinations have been increasingly used in many OECD countries, with the average number of MRI examinations in OECD countries in 2020 having increased roughly 1.6 times over that observed in 2010. Although the average number of MRI units in OECD countries in 2020 has increased about 1.5 times over that in 2010, waiting times for MRI examinations have tended to be longer because the duration of MRI examinations has become longer [2]. Thus, the resultant increase in examination duration and waiting times is one of the major issues for MRI [3].

Currently, many academic institutions use 3-T rather than 1.5-T field strength to perform female pelvic MRI due to the higher signal-to-noise ratio (SNR) of 3-T systems, although no clinical advantages of 3-T systems have been demonstrated [4–15]. In some institutions and guidelines, the use of the 1.5-T systems is preferred over 3-T systems in pregnant patients [16]. Moreover, 1.5-T systems are considered as valuable as 3-T systems on whole-body imaging, as well as diffusion-weighted image (DWI) alone and routine MR imaging with DWI, although 3-T have been suggested as more useful than 1.5-T systems for prostate MRI without endorectal coils [6, 17, 18].

Since 2019, the efficacy of compressed sensing (CS) and deep learning reconstruction (DLR) has been tested and compared with that of routinely applied parallel imaging (PI) using 3-T systems not only for female pelvic studies, but also for other body areas or clinical purposes [15, 19–32]. Although T2-weighted sequences are considered the most important ones for routine pelvic MRI, other sequences such as those T1-weighted, are also used. To date, no major studies have assessed the efficacy of CS and DLR for female pelvic MRI in comparison with that of PI at 1.5 T. The purpose of this study was to determine the efficacy of CS and DLR for shorter examination time and better image quality compared with those obtainable with PI using female pelvic 1.5-T MRI.

## Methods

### Protocol, support, and funding

This retrospective study was approved by the Institutional Review Board of Fujita Health University Hospital (HM20-075, date of approval 21st July 2020) and technically and financially supported by Canon Medical Systems Corporation (Otawara, Japan). The IRB waived the need for written informed consent from each of the subjects in this study. Four of the authors are employees of Canon Medical Systems Corporation (K.Y., N.Y., M.I., and M.Y.) but did not have control over any of the data used in this study.

#### Subjects

**Fig. 1:**
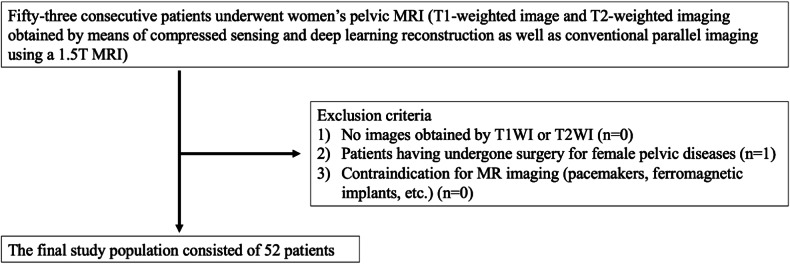
Patient flowchart showing inclusions and exclusions

Between January and March 2021, 53 consecutive patients with female pelvic diseases (mean age 45 years, range 22–85 years) underwent 1.5-T pelvic MRI using CS and DLR, as well as conventional PI. The exclusion criteria were: (1) no availability of T1-weighted images (T1WI) and T2-weighted images (T2WI); (2) previous surgery for female pelvic diseases; and (3) contraindications to MRI (such as pacemakers, ferromagnetic implants, etc.). One patient was excluded due to criterion 1. Finally, 52 consecutive patients with female pelvic diseases (mean age 44 years, range 22–85 years) were included. The patient selection flowchart is shown in Fig. 1.

### MRI protocol

All examinations were performed with a 1.5-T system (Vantage Orian; Canon Medical Systems Corporation, Otawara, Japan) using a multiple phased-array surface coil: 16 channels for Atlas SPEEDER Body and 32 channels for Atlas SPEEDER Spine (Canon Medical Systems). To determine the utility of CS and DLR, each subject was studied with T1- and T2-weighted fast spin-echo sequences using CS and conventional PI. CS data was then reconstructed with and without DLR. In this study, CS (Compressed SPEEDER, Canon Medical Systems) was used with a reduction factor of 2 (*i.e*., k-space 50% undersampling) for both T1- and T2-weighted sequences.

For T1-weighted sequences, the scan parameters were: repetition time 515 ms; echo time 15 ms; excitation/refocus flip angles 90°/180°; plane: axial; acquisition matrix 224 × 256; reconstruction matrix 512 × 512; slice thickness 4 m; interslice gap 0.4 mm; field of view 250 × 250 mm^2^. For T2-weighed sequences, they were 5,096 ms, 93.5 ms, 90°/160°, sagittal, 352 × 352, 704 × 704, 4 mm, 0.4 mm, 250 × 250 mm^2^, respectively. The number of excitations (NEX) for both T1- and T2-weighted sequences with CS was 1, while it was 2 for those with PI. All CS data was reconstructed with and without DLR.

Details of DLR have been presented in previous papers [19–22, 24–27, 29–32]. In brief, the DLR method provided by Canon Medical Systems (Advanced intelligent Clear IQ Engine-AiCE) and used in this study is a convolutional neural network. In the study presented here, times for each data acquisition and reconstruction procedure were combined and recorded as “examination time” for each sequence for each patient.

### Image analysis

All quantitative and qualitative assessments of image quality were performed on a commercially available workstation (Vitrea version 7.4, Canon Medical Systems).

#### Quantitative analysis

A board-certified female pelvic radiologist (T.U.) with 12 years of experience performed region of interest (ROI)-based measurements. Circular ROIs of 5–10 mm in diameter were placed over the myometrium, iliac muscle, multifidus muscle fat tissue of the gluteal region, and straight muscle of the abdomen on T1WI and T2WI for each patient. As for quantitative image indexes, SNR and contrast-to-noise ratio (CNR) were calculated by means of previously published formulas [19, 33–36]. For SNR, they were the following:

$${{{\rm{SNR}}}}_{{{\rm{T}}}1{{\rm{WI\; axial}}}}={{{\rm{SI}}}}_{{{\rm{Iliac\; muscle}}}}/{{{\rm{SD}}}}_{{{\rm{Iliac\; muscle}}}}$$

$${{{\rm{SNR}}}}_{{{\rm{T}}}2{{\rm{WI\; sagittal}}}}={{{\rm{SI}}}}_{{{\rm{Multifidus\; muscle}}}}/{{{\rm{SD}}}}_{{{\rm{Multifidus\; muscle}}}}$$

where SI is signal intensity and SD is the standard deviation.

For T1-weighted axial imaging, SI_Iliac muscle_ is the averaged SI of the iliac muscle within the ROI placed on either side, and SD_Iliac muscle_ is the average SD of the iliac muscle within the ROI on either side. For T2-weighted sagittal imaging, SI_Multifidus muscle_ is the average SI of the multifidus muscle within the ROI placed on the same slice, and SD_Multifidus muscle_ is the average SD of the multifidus muscle within the ROI on the same slice.

For CNR, the formulas were the following:

$${{{\rm{CNR}}}}_{{{\rm{T}}}1{{{\rm{WI}}}\; {{\rm{axial}}}}}=({{{\rm{SI}}}}_{{{{\rm{Fat}}}\; {{\rm{tissue}}}\; {{\rm{of}}}\; {{\rm{gluteal}}}\; {{\rm{region}}}}}{{{-}}}{{{\rm{SI}}}}_{{{{\rm{Iliac}}}\; {{\rm{muscle}}}}})/{{{\rm{SD}}}}_{{{{\rm{Iliac}}}\; {{\rm{muscle}}}}}$$

$${{{\rm{CNR}}}}_{{{\rm{T}}}2{{{\rm{WI}}}\; {{\rm{sagittal}}}}}= 	 \,({{{\rm{SI}}}}_{{{\rm{Myometrium}}}}{{{-}}}{{{\rm{SI}}}}_{{{{\rm{Straight}}}\; {{\rm{muscle}}}\; {{\rm{of}}}\; {{\rm{abdomen}}}}}) \\ 	 /{{{\rm{SD}}}}_{{{{\rm{Straight}}}\; {{\rm{muscle}}}\; {{\rm{of}}}\; {{\rm{abdomen}}}}}$$

For T1-weighted axial imaging, SI_Iliac muscle_ and SD_Iliac muscle_ are, respectively, the SI and SD of the iliac muscle within the ROI placed on the same slice as the previously determined SI_Fat tissue of the gluteal region_. For T2-weighted sagittal imaging, the SI_Straight muscle of the abdomen_ and SD_Straight muscle of the abdomen_ are, respectively, the SI and SD of the straight muscle of the abdomen within the ROI placed on the same slice as the previously determined SI_Myometrium_.

#### Qualitative analysis

For qualitative assessment of image quality, the same female pelvic radiologist (T.U.) who performed quantitative assessment and a board-certified radiologist with 28 years experience (Y.O.) independently visually evaluated overall image quality (OIQ), artifacts, and diagnostic confidence level (DCL) by using a 5-point scoring system. OIQ was rated as: 1, poor; 2, fair; 3, moderate; 4, good; and 5, excellent. Artifacts were scored as: 1, no visualization of artifacts; 2, visualization of few artifacts; 3, visualization of a few artifacts; 4, visualization of several artifacts; and 5, visualization of marked number of artifacts. DCL was rated as with: 1, ≤ 20% confidence (very unsure); 2, 21–40% confidence; 3, 41–60% confidence; 4, 61–80% confidence; and 5, 81–100% confidence (highly confident). All final visual scores for each patient were determined by the consensus of the two readers.

### Statistical analysis

For determining the efficacy of CS for reducing examination time while maintaining or improving image quality by DLR, mean examination times for T1WI and T2WI were compared for CS with and without DLR and for PI by means of Tukey’s honestly significant difference—HSD test. Examination time was determined in this study as the combined acquisition and reconstruction times. To compare quantitative image quality indexes for the three methods, SNRs and CNRs of T1WI and T2WI were compared for CS with and without DLR and for PI by means of Tukey’s test.

Interobserver agreement for each qualitative index was evaluated by using Cohen’s linearly weighted *κ* statistics and χ^2^ test. Interobserver agreements were considered as poor for *κ* < 0.21, fair for *κ* = 0.21–0.40, moderate for *κ* = 0.41–0.60, substantial for *κ* = 0.61–0.80, and excellent for *κ* = 0.81–1.00. Next, each of the qualitative indexes for the three methods was also compared by means of the Wilcoxon signed—rank test.

A *p-*value less than 0.05 was considered significant for all statistical analyses. All statistical analyses were performed with commercially available statistical software (JMP 14.2, SAS Institute Japan, Tokyo, Japan).

## Results

**Fig. 2:**
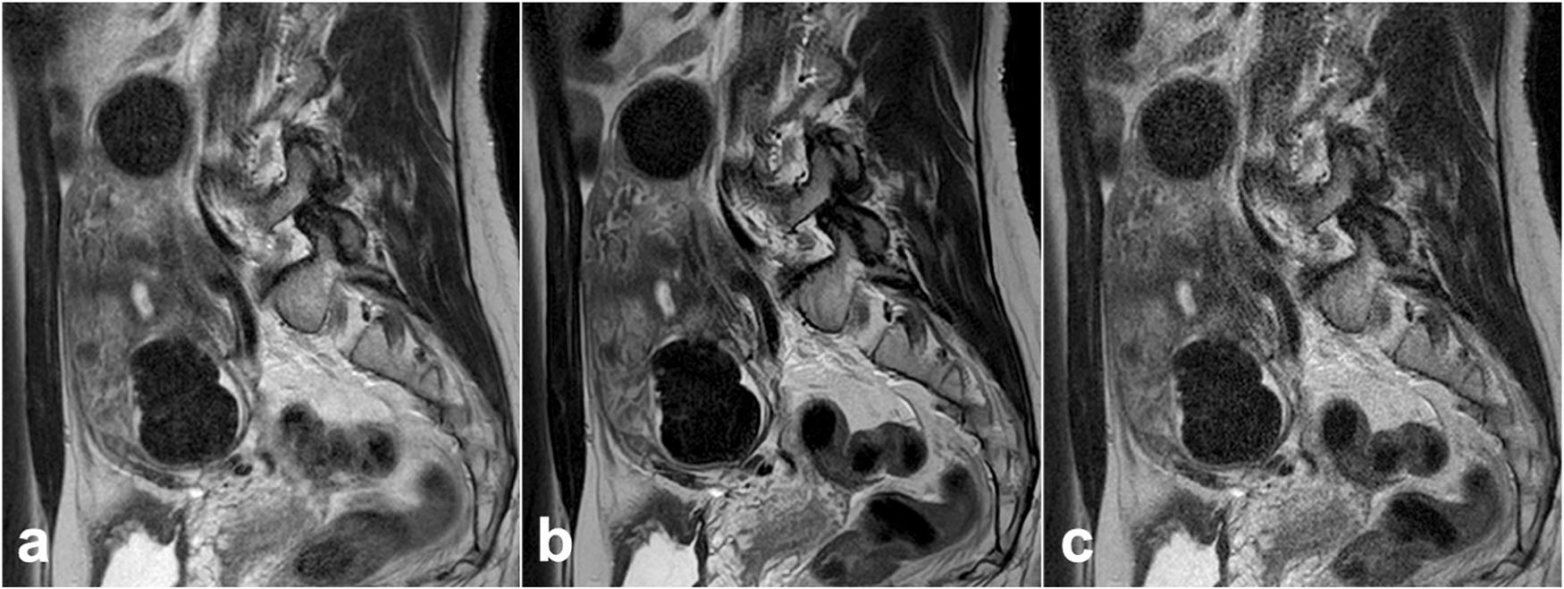
A 50-year-old female patient with uterine myoma on T2WI obtained with PI (**a**), CS with DLR (**b**), and CS without DLR (**c**). Examination times were 285 s, 107 s, and 103 s, respectively. CS with DLR (**b**) reduced image noise and showed uterine myoma, as well as normal structure more clearly than PI (**a**) or CS without DLR (**c**)

**Fig. 3:**
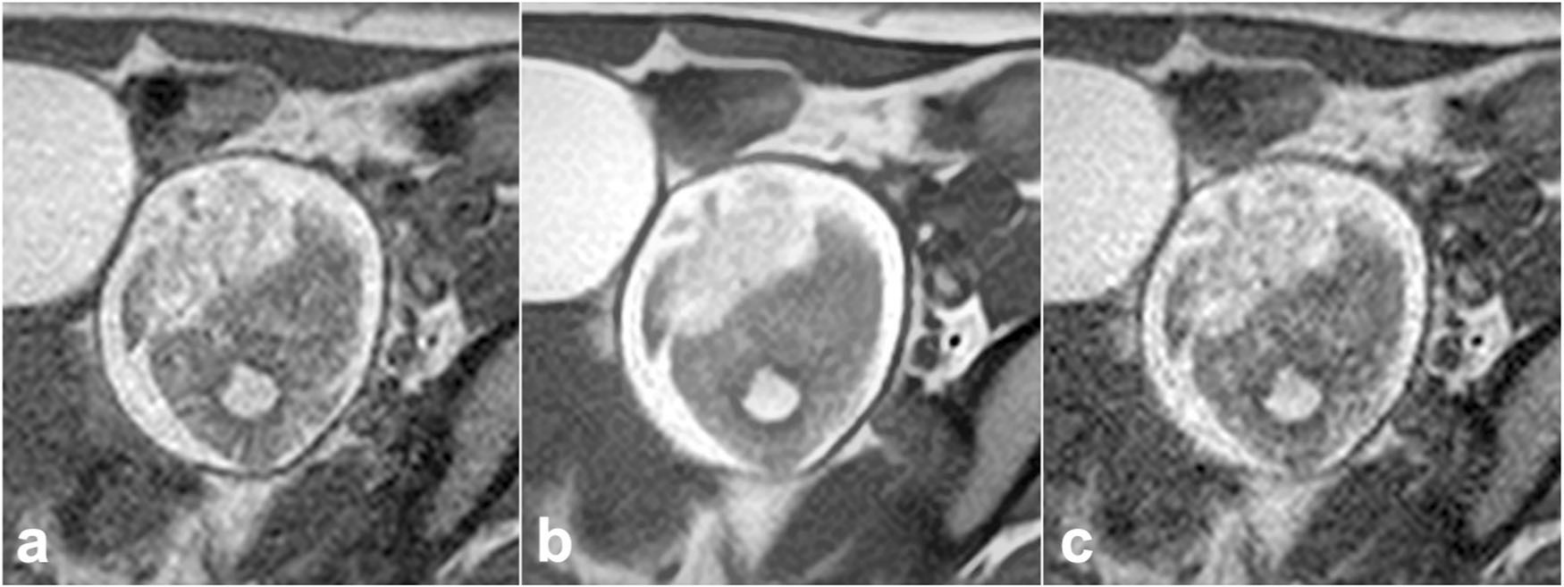
A 32-year-old female patient with ovarian mature teratoma on T1WI obtained with PI (**a**), CS with DLR (**b**), and CS without DLR (**c**). All images clearly display the ovarian mature teratoma. Examination times were 243 s, 62 s, and 58 s, respectively. CS with DLR (**b**) reduced image noise and showed the ovarian mature teratoma more clearly than does conventional PI (**a**) or CS without DLR (**c**)

All the patients had clinically or pathologically diagnosed uterine and ovarian diseases consisting of 57 benign uterine or ovarian lesions, eight malignant uterine or ovarian lesions, and three others (one pelvic abscess, two functional ovarian cysts). Details of patient characteristics are shown in Table 1. A representative case is shown in Figs. 2 and 3.

**Table 1:** Patient characteristics (histopathology of pelvic disease) of the 52 consecutive female patients included in this study (mean age 44 years, range 22–85 years)

Histopathology		Number, (%)
Benign tumors: 57 (83.8%)	Uterine myoma	23 (33.8%)
	Ovarian tumor	8 (11.8%)
	Ovarian endometriotic cyst	14 (20.6%)
	Ovarian mature teratoma	5 (7.3%)
	Hematosalpinx	1 (1.5%)
	Hydrosalpinx	2 (2.9%)
	Deep endometriosis	1 (1.5%)
	Endometrial polyp	2 (2.9%)
	Uterine adenomyosis	1 (1.5%)
Malignant tumors: 8 (11.8%)	Uterine leiomyosarcoma	1 (1.5%)
	Uterine cervical cancer	4 (5.9%)
	Uterine corpus cancer	2 (2.9%)
	Metastatic ovarian cancer	1 (1.5%)
Others: 3 (4.4%)	Pelvic abscess	1 (1.5%)
	Functional ovarian cyst	2 (2.9%)

Table 2 shows a comparison of the results for mean examination time and quantitative image quality for the three methods on T1WI and T2WI. Comparison of mean examination times showed those for CS with and without DLR on T1WI and T2WI were significantly shorter than those for PI (*p* < 0.001).

**Table 2:** Comparison of examination time and quantitative image quality for the three methods for T1WI and T2WI

Method		Examination times, (s)			SNR			CNR		
		Mean ± SD	95% CI	*p*-value *versus* CS with DLR	Mean ± SD	95% CI	*p*-value *versus* CS with DLR	Mean ± SD	95% CI	*p*-value *versus* CS with DLR
T1WI	PI	239.4 ± 18.6^a^	234.2–244.5	< 0.001	10.1 ± 2.6^a^	9.4–10.9	< 0.001	13.2 ± 2.7^a^	12.4–13.9	< 0.001
	CS with DLR	68.7 ± 5.7	67.1–70.2	−	18.9 ± 4.9	17.5–20.3	–	19.3 ± 7.2	17.3–21.3	–
	CS without DLR	64.4 ± 4.9	63.0–65.7	0.516	8.5 ± 2.4^a^	7.9–9.2	< 0.001	9.9 ± 3.3^a^	9.0–10.9	< 0.001
T2WI	PI	289.2 ± 18.1^a^	284.2–294.2	< 0.001	5.5 ± 1.2^a^	5.1–5.9	< 0.001	1.6 ± 0.7^a^	1.4–1.9	0.003
	CS with DLR	109.1 ± 7.2	107.1–111.0	–	7.0 ± 1.3	6.7–7.4	–	2.1 ± 0.8	1.8–2.3	–
	CS without DLR	105.0 ± 0.2	103.1–106.9	0.578	3.9 ± 0.7^a^	3.7–4.1	< 0.001	1.4 ± 0.5^a^	1.2–1.5	< 0.001

*CI* Confidence interval, *CS* Compressed sensing, *DLR* Deep learning reconstruction, *PI* Parallel imaging, *SD* Standard deviation, *T1WI* T1-weighted images, *T2WI* T2-weighted images

^a^ Significantly different from CS with DLR (*p* < 0.05)

Comparison of SNRs for T1WI and T2WI for the three methods demonstrated that SNRs of T1WI and T2WI obtained by using CS with DLR were significantly higher than without the use of DLR (*p* < 0.001). Moreover, SNRs obtained by using CS with DLR were significantly higher than those using PI for both T1WI (*p* < 0.001) and T2WI (*p* = 0.01).

Comparisons of CNRs among the three methods showed that CNRs obtained by using CS with DLR were significantly higher than those obtained without DLR for both T1WI (*p* < 0.001) and T2WI (*p* < 0.001), as they were for results obtained with PI (*p* < 0.001 and *p* = 0.003, respectively).

Tables 3 and 4 show the results for interobserver agreement and qualitative image quality index for T1WI and T2WI. Interobserver agreements for OIQs, artifacts, and DCLs resulting from the use of all methods on T1WI and T2WI were rated as ‘substantial’ or ‘excellent’ (*p* < 0.001).

**Table 3:** OIQ, artifacts, and DCL for the three methods on T1WI

Method	Reader	OIQ			Artifacts			DCL		
		*κ* value	*p*-value	Median, (IQR)	*κ* value	*p*-value	Median, (IQR)	*κ* value	*p*-value	Median, (IQR)
PI	Consensus	–		4 (3.25–4)^a^	–		2 (2–2.75)^a^	–		3 (1–5)
	Reader 1	0.84	< 0.001	4 (3.25–4)	0.84	< 0.001	2 (2–2.75)	0.99	< 0.001	3 (1–5)
	Reader 2			4 (3–4)			2 (2–3)			3 (1–5)
CS with DLR	Consensus	–		5 (5–5)	–		1 (1–1)	–		1.5 (1–5)
	Reader 1	0.71	< 0.001	5 (5–5)	0.71	< 0.001	1 (1–1)	0.99	< 0.001	1.5 (1–5)
	Reader 2			5 (5–5)			1 (1–1)			2 (1–5)
CS without DLR	Consensus	–		4 (3–4)^a^	–		2 (2–3)^a^	–		2 (1–5)
	Reader 1	0.84	< 0.001	4 (3–4)	0.84	< 0.001	2 (2–3)	0.99	< 0.001	2 (1–5)
	Reader 2			4 (3–4)			2 (2–3)			2 (1–5)

*CS* Compressed sensing, *DLR* Deep learning reconstruction, *IQR* Interquartile range, *PI* Parallel imaging

^a^ Significantly different from CS with DLR (*p* < 0.05)

**Table 4:** OIQ, artifacts, and DCL for the three methods on T2WI

Method	Reader	OIQ			Artifacts			DCL		
		*κ* value	*p*-value	Median, (IQR)	*κ* value	*p*-value	Median, (IQR)	*κ* value	*p*-value	Median, (IQR)
PI	Consensus	–		4 (4–5)^a^	–		2 (1–2)^a^	–		5 (4–5)^a^
	Reader 1	0.83	< 0.001	4 (4–5)	0.88	< 0.001	2 (1–2)	0.83	< 0.001	4 (4–5)
	Reader 2			4 (4–5)			2 (1–2)			4 (4–5)
CS with DLR	Consensus	–		5 (5–5)	–		1 (1–1)	–		5 (5–5)
	Reader 1	0.79	< 0.001	5 (5–5)	0.79	< 0.001	1 (1–1)	0.79	< 0.001	5 (5–5)
	Reader 2			5 (5–5)			1 (1–1)			5 (5–5)
CS without DLR	Consensus	–		4 (4–5)^a^	–		2 (1–2)^a^	–		5 (4.25–5)^a^
	Reader 1	0.94	< 0.001	4 (4–5)	0.95	< 0.001	2 (1–2)	0.94	< 0.001	4 (4–5)
	Reader 2			4 (4–5)			2 (1–2)			4 (4–5)

*CS* Compressed sensing, *DLR* Deep learning reconstruction, *IQR* Interquartile range, *PI* Parallel imaging

^a^ Significantly different from CS with DLR (*p* < 0.05)

Comparison among the three methods of each of the OIQs for T1WI and T2WI showed that OIQs of T1WI and T2WI obtained by using CS with DLR were significantly higher than those obtained by using PI or CS without DLR (*p* < 0.001). Furthermore, a comparison among the three methods of artifacts for T1WI and T2WI showed that for T1WI and T2WI obtained by using CS with DLR, there were significantly fewer than by using PI or CS without DLR (*p* < 0.001). Finally, a comparison of DCLs among the three methods showed that DCLs of T2WI obtained using CS with DLR were significantly higher than those using PI or CS without DLR (*p* < 0.001).

## Discussion

Our findings demonstrate CS and DLR, when compared with PI, can improve image quality, including CNR, while shortening examination time for not only T2WI, but also T1WI for female pelvic MRI using a 1.5-T system. To the best of our knowledge, this study is the first to demonstrate the clinical potentials of CS and DLR for female pelvic MRI in comparison with conventional PI or CS alone using a 1.5-T MR system.

Comparisons of mean examination times for T1WI and T2WI showed those using CS were significantly shorter than for PI, regardless of whether each of the MR images was obtained by using CS with or without DLR. These findings are compatible with those previously published for using a 3-T system [19–21, 29].

Comparisons of quantitative image quality showed SNRs and CNRs of T1WI and T2WI obtained by using CS with DLR were significantly higher than those of others. These findings suggest DLR reduces image noise and improves SNRs and CNRs when T1WI and T2WI of patients with female pelvic diseases are obtained by using CS. Moreover, with the use of CS and a reduction in NEX, DLR is potentially superior to PI for improving image noise, SNRs, and CNRs on T1WI and T2WI obtained by CS, as compared with PI, whose NEX was double. In view of the results of this, as well as previous studies using a 3-T MR system, CS should thus be better used in combination with DLR for routine clinical practice rather than PI for female pelvic MR imaging using not only 1.5-T, but also 3-T systems [20, 21, 27, 30–32].

Interobserver agreements for all qualitative indexes on T1WI and T2WI obtained with the three methods were rated as ‘substantial’ or ‘excellent’. Our evaluation for each index can thus be considered reproducible [37].

Comparisons of all qualitative image qualities show that OIQ and artifacts on T1WIs obtained by using CS with DLR were superior to those on other T1WIs, although there were no significant differences in DCL evaluation among the three methods. Moreover, each of the qualitative indexes for T2WI obtained by means of CS with DLR was significantly higher than that of other T2WIs. These results are compatible with the quantitative image quality analysis results in this study, which could have been anticipated in view of previously published results when using 3-T systems [19–22, 24, 29]. In comparison with currently used conventional PI for female pelvic MRI, DLR may well be more effective for better quantitative and qualitative image quality on T1WI and T2WI, while CS should be used in combination with DLR for shorter examination times when using a 1.5-T MR system in routine clinical practice.

There were several limitations to this study. First, the number of subjects was limited, and their underlying pathological and clinical conditions varied; in addition, the number of malignant lesions examined in this study was limited. Second, we used a different NEX for CS for comparison with conventional PI. The NEX for conventional PI was 2 (NEX 1 could not be used for conventional PI due to SNR limitations of the MRI system and sequences, *i.e*., sensitivity and noise performance of acquisition and receive coil). Therefore, this difference could have affected our results for comparison mean examination times. Third, we tested CS and DLR to obtain T1WI and T2WI on a 1.5-T system but did not use another field strength. Moreover, diagnostic performances by the three methods were not compared. Fourth, this study only evaluated unenhanced T1WI and T2WI and did not assess other important sequences such as diffusion-weighted imaging or dynamic contrast-enhanced imaging. Fifth, the reproducibility of the provided results was not investigated for other MRI systems and CS and DLR techniques from other vendors. Sixth, in this study, we calculated SNR as the mean SI within ROI divided by SD within ROI according to past literature [19, 33–36]. However, there are different equations for assessing SNR in other papers. Therefore, our results had some biases based on differences in SNR equations as compared with other methods. Further investigations are warranted to determine the real significance of these techniques for routine female pelvic MRIs.

In conclusion, CS and DLR are more effective than PI for shortening examination time and improving the image quality of female pelvic MRI on a 1.5-T system. The addition of DLR renders CS even more effective for improving image quality and improving not only SNR, but also CNR on T1WI and T2WI in this setting.

## Data Availability

The datasets used and/or analyzed during the current study are available from the corresponding author upon reasonable request.

## Abbreviations

CNR: Contrast-to-noise ratio
CS: Compressed sensing
DCL: Diagnostic confidence level
DLR: Deep learning reconstruction
DWI: Diffusion-weighted image
NEX: Number of excitations
OIQ: Overall image quality
PI: Parallel imaging
ROI: Region of interest
SD: Standard deviation
SI: Signal intensity
SNR: Signal-to-noise ratio
T1WI: T1-weighted images
T2WI: T2-weighted images

## Declarations

### Guarantor

This study was approved by the Institutional Review Board (IRB) of Fujita Health University Hospital. The IRB waived the need for written informed consent from each of the subjects in this study because of the retrospective study.
