# Correction: Efficacy of compressed sensing and deep learning reconstruction for adult female pelvic MRI at 1.5 T

**DOI:** 10.1186/s41747-024-00521-6

**Published:** 2024-10-16

**Authors:** Takahiro Ueda, Kaori Yamamoto, Natsuka Yazawa, Ikki Tozawa, Masato Ikedo, Masao Yui, Hiroyuki Nagata, Masahiko Nomura, Yoshiyuki Ozawa, Yoshiharu Ohno

**Affiliations:** 1 Department of Diagnostic Radiology, Fujita Health University School of Medicine, Toyoake, Japan; 2 Canon Medical Systems Corporation, Otawara, Japan; 3 Department of Radiology, Fujita Health University Bantane Hospital, Nagoya, Japan; 4 Joint Research Laboratory of Advanced Medical Imaging, Fujita Health University School of Medicine, Toyoake, Japan

**Correction to: European Radiology Experimental**

10.1186/s41747-024-00506-5, published online 10 September 2024

In this article, the affiliation details for authors Masato Ikedo and Masao Yui were incorrectly given as ‘Joint Research Laboratory of Advanced Medical Imaging, Fujita Health University School of Medicine, Toyoake, Japan’ but should have been ‘Canon Medical Systems Corporation, Otawara, Japan’.

Furthermore, in the Graphical Abstract, it incorrectly stated that the top row figures are T1-weighted and the bottom row figures are T2-weighted. The correct statement is that the top row figures are ‘T2-weighted’ and the bottom row figures are ‘T1-weighted’.

The corrected Graphical Abstract can be seen below:

**Figure:**
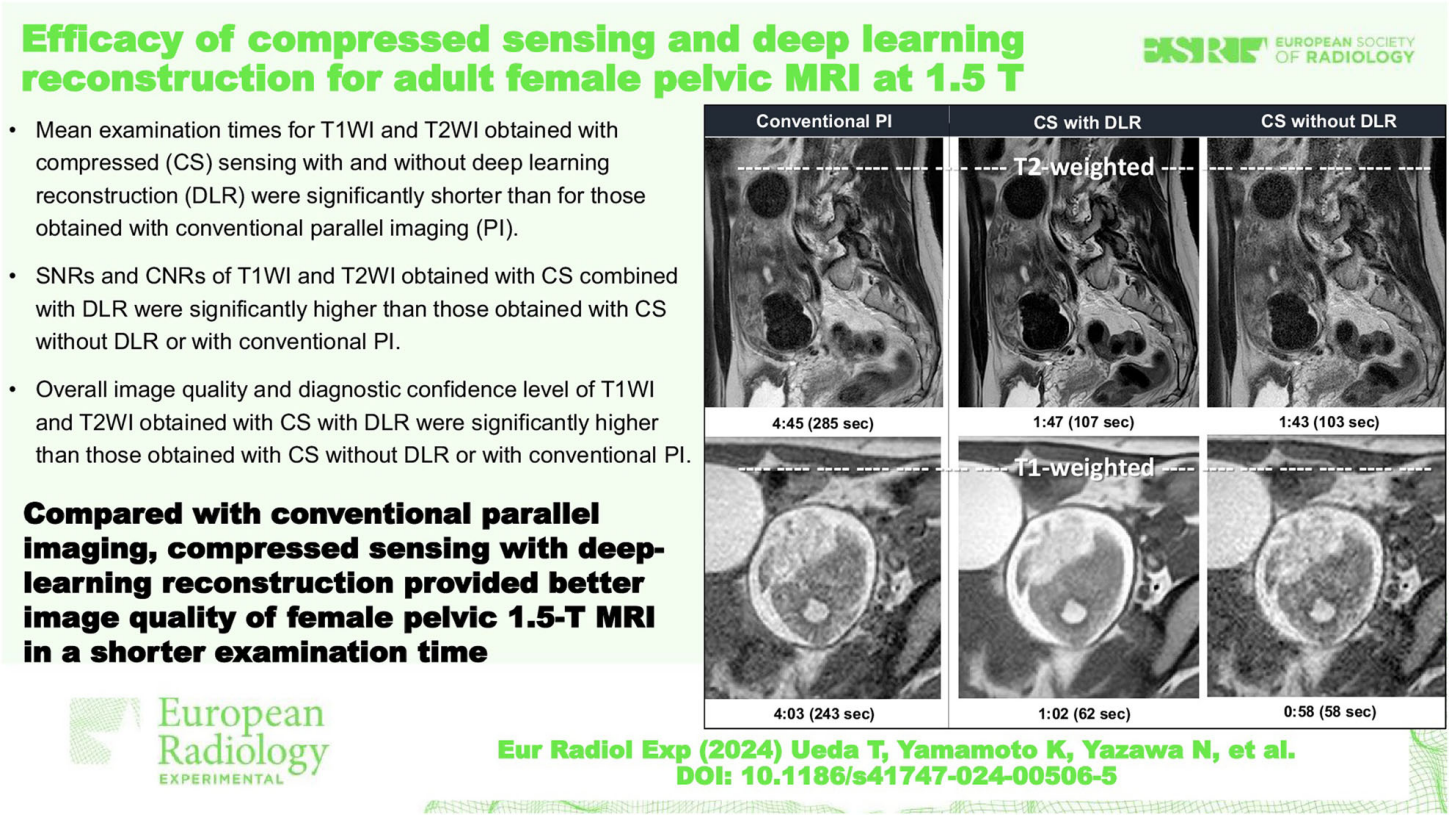

The original article has been corrected.

